# Predictive performance of count regression models versus machine learning techniques: A comparative analysis using an automobile insurance claims frequency dataset

**DOI:** 10.1371/journal.pone.0314975

**Published:** 2024-12-31

**Authors:** Gadir Alomair

**Affiliations:** Department of Quantitative Methods, School of Business, King Faisal University, Al-Ahsa, Saudi Arabia; Cairo University, EGYPT

## Abstract

Accurate forecasting of claim frequency in automobile insurance is essential for insurers to assess risks effectively and establish appropriate pricing policies. Traditional methods typically rely on a Poisson distribution for modeling claim counts; however, this approach can be inadequate due to frequent zero-claim periods, leading to zero inflation in the data. Zero inflation occurs when more zeros are observed than expected under standard Poisson or negative binomial (NB) models. While machine learning (ML) techniques have been explored for predictive analytics in other contexts, their application to zero-inflated insurance data remains limited. This study investigates the utility of ML in improving forecast accuracy under conditions of zero-inflation, a data characteristic common in automobile insurance. The research involved a comparative evaluation of several models, including Poisson, NB, zero-inflated Poisson (ZIP), hurdle Poisson, zero-inflated negative binomial (ZINB), hurdle negative binomial, random forest (RF), support vector machine (SVM), and artificial neural network (ANN) on an insurance dataset. The performance of these models was assessed using mean absolute error. The results reveal that the SVM model outperforms others in predictive accuracy, particularly in handling zero-inflation, followed by the ZIP and ZINB models. In contrast, the traditional Poisson and NB models showed lower predictive capabilities. By addressing the challenge of zero-inflation in automobile claim data, this study offers insights into improving the accuracy of claim frequency predictions. Although this study is based on a single dataset, the findings provide valuable perspectives on enhancing prediction accuracy and improving risk management practices in the insurance industry.

## 1. Introduction

Automobile insurance is an essential component of modern society, providing financial protection for individuals against various types of loss associated with automobiles, such as vehicle damage, theft, or costs incurred due to accidents. A key aspect of this industry is the accurate prediction of claims frequency, which significantly impacts an insurer’s profitability. This task is further complicated by the presence of zero-inflation in the data, where a disproportionate number of policyholders do not submit claims during a given period, leading to an over-abundance of zero counts. Claims frequency refers to the number of claims filed by policyholders over a specific period. Accurately predicting claims frequency is crucial for actuaries and data scientists in insurance companies, guiding their underwriting decisions and premium pricing strategies [1–3].

Traditional count models, like Poisson and negative binomial, are commonly used for claims frequency prediction due to their suitability for count data. However, they often struggle with overdispersion and zero-inflation, where the variance exceeds the mean or there is an overabundance of zeros [4–8]. To address these issues, zero-inflated and hurdle models have been developed, offering more flexibility by accounting for the excess zeros [9–11]. Studies by Zhang et al. [12], as well as Erdemir et al. [13], offer valuable insights into multivariate count models for handling zero inflation in insurance data, marking a significant advancement in the field.

With the rise of machine learning (ML), more sophisticated models such as Random Forest (RF), Support Vector Machines (SVM), and Artificial Neural Networks (ANN) have been applied to count data. These models can capture complex, nonlinear relationships, making them well-suited for large, high-dimensional datasets. Several studies have explored the predictive performance of ML models for count data [14–18]. However, a direct comparison of these models with traditional count models on zero-inflated automobile claims data remains limited.

Recent research efforts have begun to compare the performance of these ML models in the field of insurance. For instance, Poufinas et al. [19] applied a variety of machine learning models, SVM, decision trees, RF, and boosting, to forecast motor insurance claims. Their study demonstrated the importance of incorporating external factors, such as weather conditions and car sales, into claims prediction models, which can improve the accuracy of machine learning models in insurance. Furthermore, Wilson et al. [20] conducted a comparative analysis of traditional GLM and machine learning models, including gradient boosting methods (GBMs) and ANNs, for predicting loss costs in motor insurance. Their study found that a hybrid model combining GLM and ANN outperformed both individual models, pointing to the potential benefits of hybrid approaches that leverage the strengths of traditional statistical methods and modern machine learning.

Recent advancements in ML for count data have led to the development of interpretable zero-inflated neural network models. Jose et al. [21] introduced the Zero-Inflated Poisson Neural Network and the Zero-Inflated Combined Actuarial Neural Network for modeling admission rates in health insurance. Their work highlights how neural networks, when combined with actuarial models, can capture complex relationships in count data while maintaining interpretability. Similarly, So [22] explored the use of GBMs, specifically CatBoost, XGBoost, and LightGBM, for modeling zero-inflated insurance claims data. His study demonstrated that CatBoost outperformed the other methods in modeling claim frequency, showing its ability to handle categorical variables and zero-inflated data effectively. Furthermore, So proposed a novel zero-inflated Poisson boosted tree model, which offers significant improvements in predictive accuracy for zero-inflated datasets. These recent studies highlight the considerable advancements in machine learning techniques for count data, especially in the context of zero-inflation. Machine learning models have shown significant improvements in predictive performance by effectively handling the unique characteristics of insurance data, such as excess zeros and non-linear relationships. These models represent a significant step forward in applying ML to count data in insurance, offering both improved predictive performance and interpretability.

Despite the growing body of research on ML models in insurance contexts, there has been limited exploration of how these techniques perform in the presence of zero-inflation, a common feature of automobile insurance claims data comprehensively compared to traditional statistical models.This study seeks to bridge the knowledge gap by conducting a thorough comparison of traditional and ML models applied to claims frequency prediction in automobile insurance, with a particular focus on handling zero-inflation. This comparison is vital for understanding the strengths and limitations of each modeling approach in the context of contemporary challenges faced by insurers. Additionally, the outcomes of this study hold practical significance. With the insurance sector operating on thin margins, the ability to predict claims frequency more accurately can lead to enhanced profitability through better risk assessment and premium pricing. Moreover, by identifying the most effective predictive models, this study can aid in the development of more resilient and adaptable insurance frameworks capable of withstanding changes in risk landscapes and policyholder behavior. By evaluating the predictive performance of a range of statistical and ML models on zero-inflated insurance data, this research provides valuable insights into the underexplored intersection between zero-inflated modeling and machine learning techniques. Although this study is based on a single dataset, the insights gained can guide future research and applications in similar contexts.

The rest of this paper is structured as follows: descriptions of the motor insurance dataset, the models utilized, and the evaluation approach are provided in the next section. Section 3 presents the results of the models’ performance, and Section 4 offers a discussion of the overall performance of the models.

## 2. Materials and methods

### 2.1 Dataset

The dataset used in this study, sourced from the SAS Enterprise Miner database [23], contains details on the frequency of insurance claims [24]. This data is utilized to assess the predictive performance of the models. The dataset includes information related to specific auto insurance policies, such as the date, frequency, and total payout of each claim throughout the policy years.

**Fig 1:**
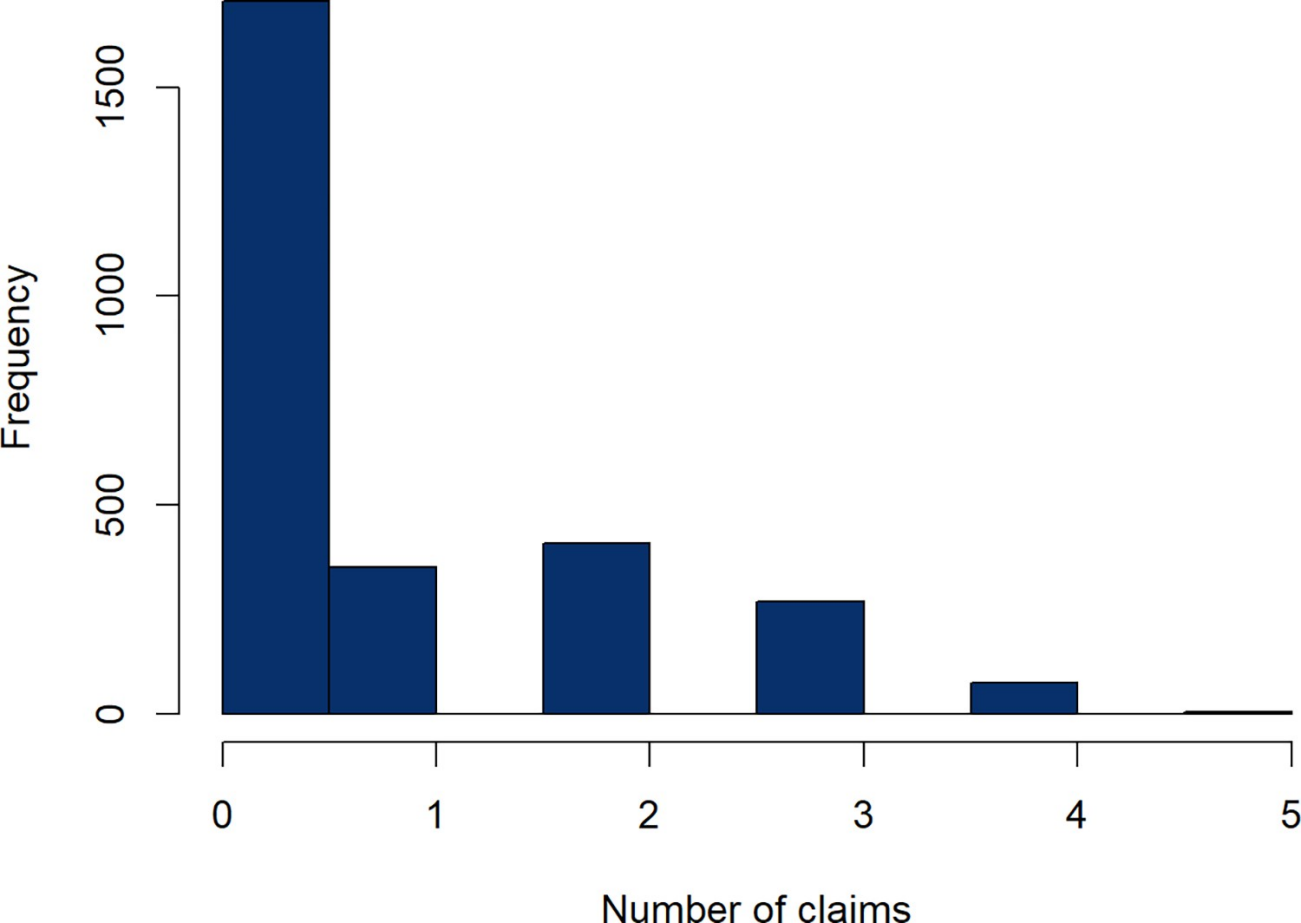
Distribution of claims in one year.

The raw dataset encompasses 10,303 observations across 33 variables. However, due to the prevalence of incomplete records, only those policyholders who maintained their contracts in the most recent year are considered. The cleaned SAS dataset consists of 2,812 complete records from the 3,712 new customers of this insurance company. The distribution of claim counts is represented in both Fig 1 and Table 1.

**Table 1:** Number of claims in one year.

Number of claims in one year, ranging from 0 to 5	Frequency	Percent (%)
0	1,706	60.7
1	351	12.5
2	408	14.5
3	268	9.5
4	74	2.6
5	5	0.2

A significant number of zero counts are observed in the claim frequency variable, likely due to a large segment of policyholders posing a low claim risk. The dataset encapsulates various factors such as claim profiles, policy specifics, driving records, and policyholders’ personal information.

The claim profile data can be used to calculate the total number of claims for each policyholder. Files containing policy details, driving history, and personal information provide insights into potential risk factors influencing claim incidents. The insurance details file includes information such as the policy number, customer ID, policy commencement date, home/work location, commute duration, and data related to the insured vehicle (e.g., value, type, usage, color).

The driving record includes a point-based system tracking the policyholder’s driving history and any instances of license revocation by government agencies over the past seven years. Infractions of traffic rules result in points being added to the policyholder’s record, with more severe violations leading to higher point penalties.

The personal particulars file consists of demographic and socioeconomic information about the policyholder, including gender, age, date of birth, marital status, number of children, annual income, employment type, and education level.

In this study, we use only the variables identified as significant by Yip et al. [25], including car usage type, policyholder income, gender, and marital status.

### 2.2 Models description

#### 2.2.1 Count regression models

The Poisson regression model is commonly used to count data, assuming that the mean is equal to the variance. The probability of observing y events (where y = 0, 1, 2,…) is given by the Poisson distribution as follows:

$$Pr(Y=y)=e^{-\lambda }\lambda^{y}/y!$$ (1)

In the regression context, the mean λ is related to the predictor variables through a log link: *ln*(*λ*) = ***Xβ***, where ***X*** represents the design matrix, and ***β*** is the parameter vector.

The NB regression model is particularly useful for over-dispersed count data (where the variance exceeds the mean). The probability of observing y events (where y = 0, 1, 2,…) is given by the NB distribution as follows:

$$Pr(Y=y)=\frac{\Gamma (y+\frac{1}{\theta })}{\Gamma (y+1)\Gamma (1/\theta )}\;{\left(\frac{1}{1+\theta \lambda }\right)}^{y}\;{\left(\frac{\theta \lambda }{1+\theta \lambda }\right)}^{\frac{1}{\theta }}$$ (2)

The mean λ is related to the predictor variables through a log link, similar to the Poisson model: *ln*(*λ*) = ***Xβ***, where *λ* is the mean count, *θ* is the dispersion parameter, ***X*** is the design matrix, and ***β*** is the parameter vector. The dispersion parameter *θ* allows for the accommodation of over-dispersion in the count data.

The distribution of excess-zero models is linked to three primary components: a random component that dictates the assumed distribution, chosen from the exponential family distributions, a systematic component illustrating the association between parameters and predictors, and a link function bridging the response mean with the systematic component. The logit function serves this purpose for the logistic component, while the log function is used for the Poisson or NB components.

Assuming the response variable is Y, excess-zero distributions with a probability π for the logistic part and a mean λ for the count part are used. The zero-inflated model’s distribution, which combines a logistic regression model for predicting a “structured zero” and count regression models for predicting counts, can be expressed as follows:

$$f_{ZI}(y;\pi ,\lambda )=\left\{ \begin{array}{c} \pi +(1-\pi )\mathrm{Pr}(K=0)\;y=0\\ (1-\pi )\mathrm{Pr}(K=y)\;y>0 \end{array}\right.$$ (3)

Similarly, the distribution of the hurdle models, which include a logistic regression model for predicting a “structured zero” (the exclusive source of zeros) and zero-truncated count regression models for predicting counts, can be expressed as follows:

$$f_{Hur}(y;\pi ,\lambda )=\left\{ \begin{array}{ll} \pi & y=0\\ (1-\pi )\frac{Pr(K=y)}{1-Pr(K=0)} & y>0 \end{array}\right.$$ (4)

Here, *K* is a random variable that can follow either Poisson or NB distributions. The following are the systematic components and link functions for the excess-zero regression models: logit(*π*) = **X**_***l***_***β***_***l***_ and ln(*λ*) = **X**_***c***_***β***_***c***_, where ***X***_***l***_ and ***β***_***l***_ are the design matrix and the parameter vector corresponding to the logistic component, respectively. Similarly, **X**_***c***_ and ***β***_***c***_ correspond to the design matrix and parameter vector for the count component.

#### 2.2.2 Machine learning models

The aim of this study is to investigate how ML regression techniques can predict claims frequency. ML techniques offer a variety of algorithms and methodologies capable of solving real-world problems. RF is an ML model designed to produce accurate predictions without overfitting the data. The RF model can be written in the form:

$$y(X)=f\{\sum_{k=1}^{K}\omega_{k}\phi (X,V_{k})\}$$ (5)

where ***V***_*k*_ is the variable chosen for splitting. Each tree in the forest is developed using a subset of *k* randomly chosen features. Although RF can handle both regression and classification problems, this study focuses exclusively on regression tasks.

SVMs are supervised ML models used for both regression and classification tasks. In this study, SVM is employed for regression purposes to predict the number of claims in automobile insurance. SVM can be written in the form:

$$y_{ij}=f(x_{ij})+\varepsilon_{ij}$$ (6)

The aim of SVM is to find a function *f*(*x*_*ij*_) that has at most *ε* deviation from the actual targets *y*_*ij*_ for all training data while remaining as flat as possible. The main parameters for SVM are the following: 1) ε, which defines the margin of tolerance; and 2) the cost parameter, which determines the trade-off between the flatness of *f*(*x*_*ij*_) and the extent to which deviations larger than ε are tolerated. It is a regularization parameter that helps control the balance between overfitting and underfitting. The default radial basis function kernel in R programming is utilized to handle the complexities and nonlinear relationships in the dataset, providing the flexibility needed to model the intricate dynamics of the data.

ANNs, inspired by biological neural networks, are a type of deep learning model. The ANN model can be represented as follows:

$$ANN=f(w,x_{ij})$$ (7)

where *w* represents the weights of the neural network. The following are the main parameters for ANNs: 1) Number of hidden layers: This impacts the level of complexity the network can handle. 2) Number of neurons in each hidden layer: This affects the model’s capacity to learn complex patterns. 3) Learning rate: This controls how much the model changes in response to the estimated error each time the model weights are updated. Following the recommendation to start with a hidden layer size between 70% and 90% of the input layer size and less than twice the input size [24], this study selects 3 hidden neurons.

### 2.3 Models evaluation

In this study, the predictive capabilities of nine models were compared. The dataset was partitioned into a training set comprising 80% of the data (2,250 observations) and a testing set comprising the remaining 20% (562 observations). This partitioning strategy ensured that the models had sufficient data to learn from while also reserving a robust portion for evaluating their predictive performance.

To address potential overfitting and ensure optimal model performance, a 5-fold cross-validation framework integrated with hyperparameter tuning was employed. This method involves partitioning the dataset into five equal-sized subsets. Each fold is used as a validation set exactly once, while the remaining four folds are used to train the model. This process is repeated five times, with a different fold serving as the validation set in each iteration. The performance metrics are then averaged across all five iterations, providing a robust estimate of the model’s generalizability and performance.

Within this cross-validation framework, hyperparameter tuning was performed using a grid search. For each fold, a search over a predefined grid of hyperparameters was conducted, and the model was evaluated on the validation set. The best hyperparameters were selected based on the lowest mean absolute error (MAE) averaged across the folds. This integrated approach ensures that the model’s performance is assessed using the optimal hyperparameters, thereby enhancing the reliability and validity of the evaluation process.

1. **Random Forest:** The number of variables tried at each split (*mtry*) was tuned with values {2, 3, 4}.
2. **Support Vector Machine:** The regularization parameter (*C*) and kernel coefficient (*sigma*) were tuned with values {0.1, 1, 10} and {0.01, 0.05, 0.1}, respectively.
3. **Artificial Neural Network:** The number of neurons in the hidden layer (*size*) and the weight decay (*decay*) were tuned with values {2, 3, 4} and {0.1, 0.01, 0.001}, respectively.

The best hyperparameters for each model are presented in Table 2.

**Table 2:** Chosen hyperparameters for machine learning models.

Model	Hyperparameter	Value
**RF**	mtry	2
**SVM**	C	10
	sigma	0.05
**ANN**	size	2
	decay	0.1

RF = random forest; SVM = support vector machine; ANN = artificial neural network.

The MAE was chosen as the evaluation metric to assess the accuracy of the models’ predictions. MAE is computed as the average of the absolute differences between the actual and predicted values:

$$MAE=(1/n)*\Sigma |y_{i}-{\hat{y}}_{i}|$$ (8)

The simplicity and direct interpretability of this metric make it particularly suitable in this context. MAE does not overly penalize larger errors and is less sensitive to outliers, which is important given the count nature and zero inflation of the data [26,27]. A model with a lower MAE is deemed superior, as it indicates that the predictions are, on average, closer to the actual values. The use of MAE as a preferred metric is supported by the work of Willmott et al. [28], who highlighted its advantages over other metrics, such as root mean squared error, for assessing average model performance.

## 3. Results

In this section, the performance of the regression models is evaluated, particularly considering the presence of zero inflation in the automobile insurance claims frequency data. A comparison is made between the performance of ML regression techniques and count regression models, and the results are shown in Table 3. The empirical insights and findings drawn from this comparison are subsequently presented.

**Table 3:** Comparison of models.

Model Type	Model	Train MAE	Test MAE
**Count Regression Models**	Poisson	0.900113	0.931333
	NB	0.900394	0.932408
	ZIP	0.897591	0.929467
	ZINB	0.897590	0.929467
	HurP	0.898361	0.929981
	HurNB	0.898359	0.929980
**Machine Learning Regression Models**	RF	0.859160	0.929868
	SVM	0.805950	0.854996
	ANN	0.893052	0.929396

NB = negative binomial; ZIP = zero-inflated Poisson; ZINB = zero-inflated negative binomial; HurP = hurdle Poisson; HurNB = hurdle negative binomial; RF = random forest; SVM = support vector machine; ANN = artificial neural network.

The performance of the count regression models was closely matched, with ZIP and ZINB models demonstrating a slight edge. MAE training and testing values for the ZIP and ZINB models were 0.9294667 and 0.9294668, respectively, indicating marginally superior performance compared to the other models. The Poisson and NB models exhibited the weakest performance among the regression models.

In terms of ML models, the SVM model demonstrated superior performance compared to RF and ANN models, with a testing MAE of 0.854996. Its training MAE of 0.805950 suggests strong generalization capabilities. The superior performance of SVM can be attributed to its ability to model complex, nonlinear relationships in the data, especially through its kernel trick, which allows it to better capture patterns in zero-inflated, over-dispersed data. Additionally, SVM’s robustness to overfitting likely contributed to its ability to generalize well, as reflected by the close training and test MAE values. The RF and ANN models exhibited similar performance, as indicated by their testing MAE values of 0.929868 and 0.929396, respectively.

**Fig 2:**
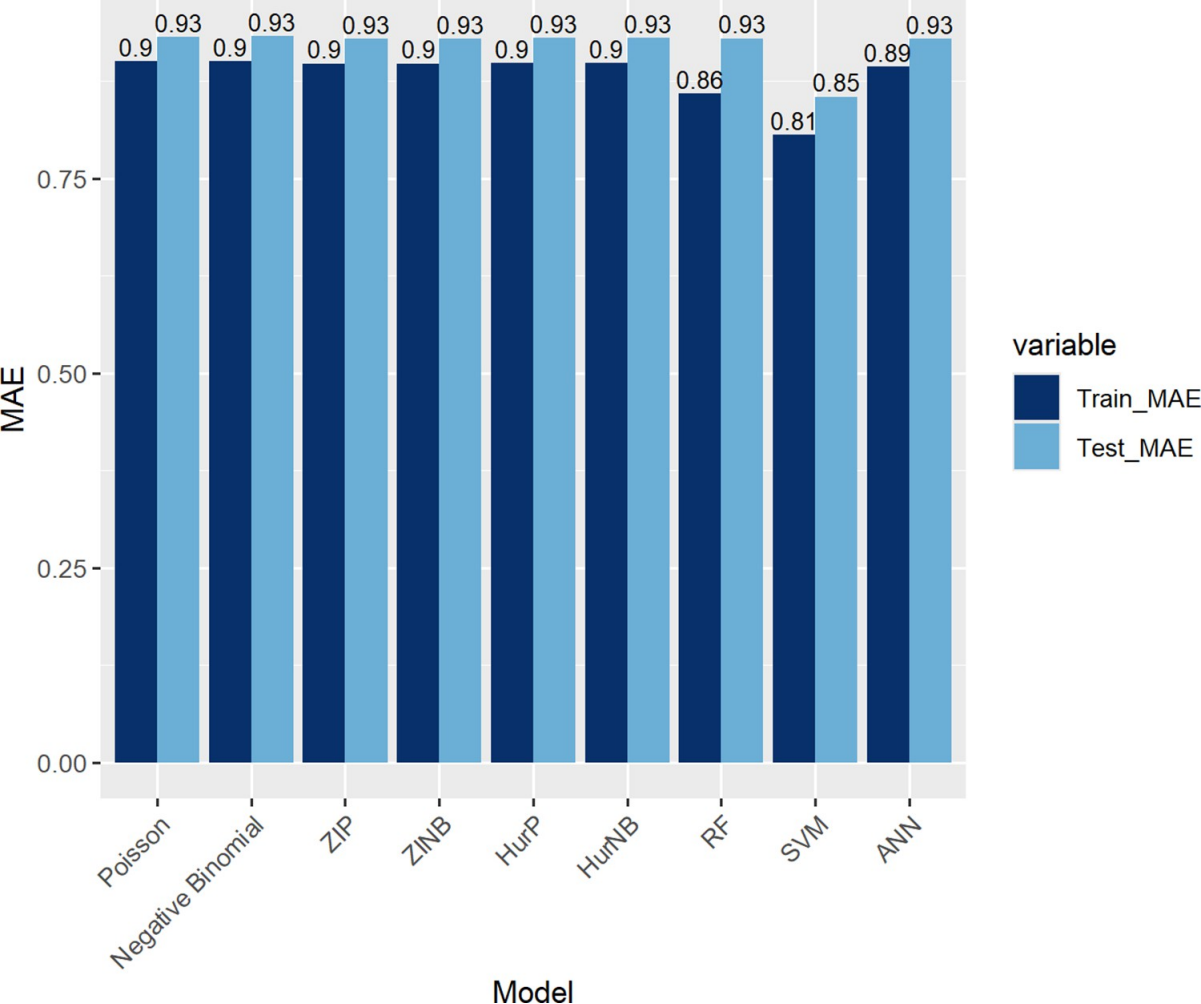
Model performance comparison.

Overall, both count regression and ML models showed that SVM outperformed all others, while Poisson and NB models demonstrated the lowest performance. The closeness of the training and testing MAE values indicates that the models do not overfit and are generalized well to new data. Regarding the remaining models, their MAE values were remarkably close, although the ZIP and ZINB models showed a slight edge. Fig 2 visually confirms the findings from Table 3, highlighting the performance differences among the models, with SVM showing the best results and Poisson and NB models performing less effectively.

**Fig 3:**
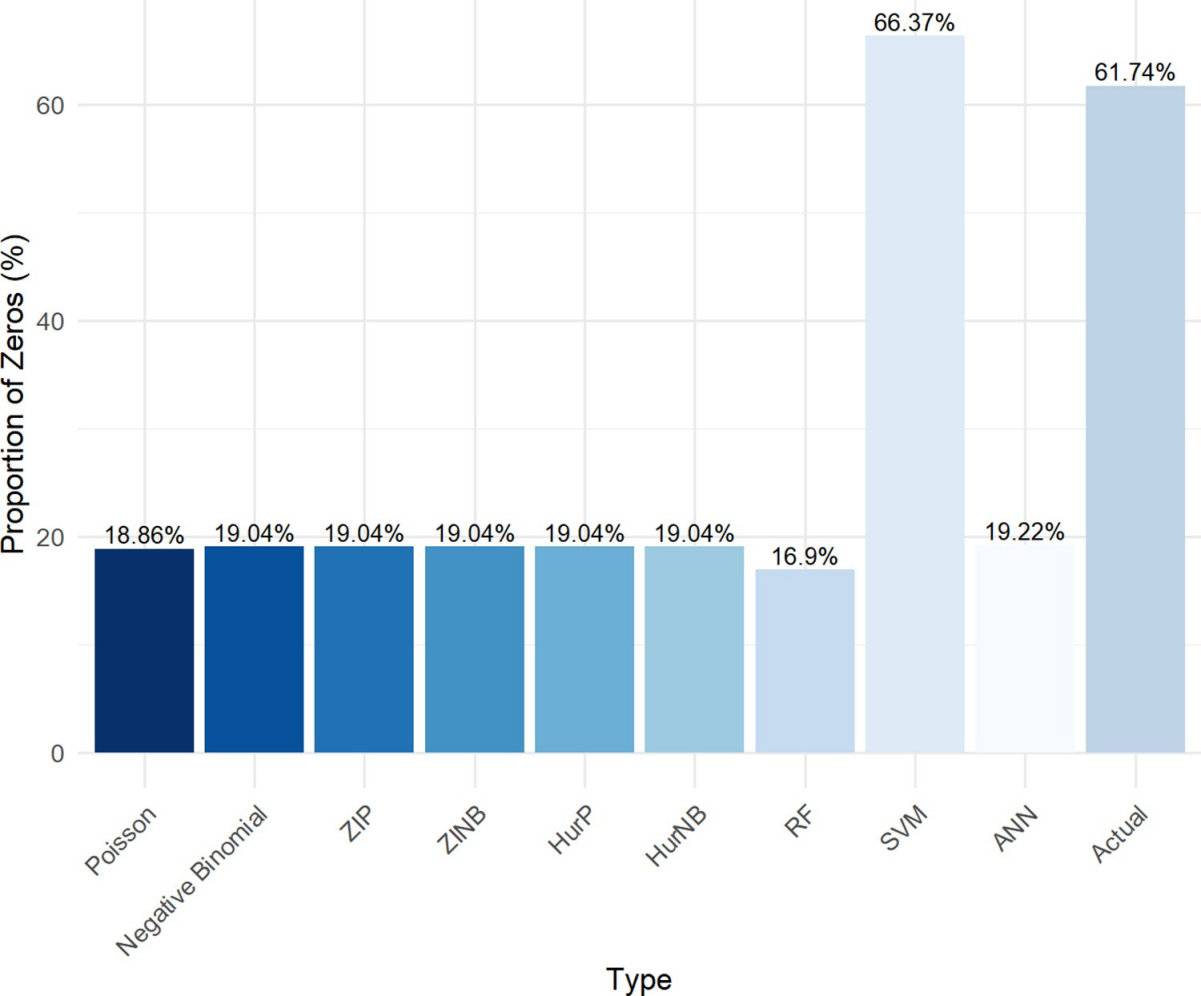
Zero inflation plot.

Fig 3 presents the zero-inflation plot, highlighting the proportion of zeros predicted by each model compared to the actual data. The SVM model predicts a zero proportion of 66.37%, closely aligning with the actual value of 61.74%. In contrast, all other models significantly underestimate the proportion of zeros. The ability of SVM to closely match the actual zero proportion further demonstrates its adaptability to zero-inflated data.

## 4. Discussion

The primary objectives of this study were to examine the predictive capabilities of count regression models and ML techniques utilizing zero-inflated claim frequency count data. A comparative analysis of the models was conducted to assess their predictive abilities using MAE as the performance metric. Traditional count regression models (Poisson, NB, ZIP, ZINB, hurdle Poisson, and hurdle negative binomial) were evaluated, along with three ML regression techniques (RF, SVM, and ANN), and their performances were contrasted against the traditional models.

The results suggest that, overall, the SVM model outperformed the other ML regression models. Notably, SVM significantly surpassed both the traditional count regression models and other ML models in terms of MAE values. This superior performance can be attributed to SVM’s ability to model complex, nonlinear relationships through its kernel function, which allows it to better capture the patterns in zero-inflated, over-dispersed data, which is particularly beneficial when working with real-world insurance data that often exhibit such patterns. Furthermore, SVMs are more flexible and do not require strict assumptions about the underlying data, unlike traditional count models. Additionally, SVM outperformed the other ML models, including RF and ANN, likely due to its ability to handle high-dimensional data without overfitting—a characteristic that makes it particularly robust for predictive modeling. This study makes a unique contribution by systematically comparing traditional statistical models with machine learning approaches, particularly in the context of zero-inflation, a challenge that is prevalent in automobile insurance data but has received limited attention in prior research.

The traditional count models, such as Poisson and NB, often struggle to capture the data-generating process accurately in the presence of excess zeros, leading to inaccurate predictions and misleading inferences. Although zero-inflated and hurdle models aim to address the issue of excess zeros, they introduce additional complexities, making them more complicated in terms of model estimation and interpretation. While the SVM slightly overestimates the proportion of zeros, it provides the most accurate prediction. Conversely, other models significantly underestimate this proportion.

While the interpretation of SVM models is less straightforward than traditional statistical models, the trade-off for improved predictive performance is often worthwhile, particularly in an insurance context where accurate claim frequency predictions can have significant financial implications. This work provides a foundation for future research exploring the integration of zero-inflated techniques with advanced machine learning models. Therefore, this study suggests that SVMs could be a valuable tool for predicting automobile insurance claims.

These findings have significant practical implications for the automobile insurance industry. By deploying SVMs, insurers can achieve more accurate predictions of claim frequencies, which can directly enhance premium pricing strategies, risk assessment, and reserve management. More accurate predictions can also lead to better resource allocation, reducing the likelihood of underpricing or overpricing insurance policies, thereby improving profitability.

Although the findings are based on a single dataset, they highlight the potential of SVMs in addressing zero-inflated insurance data. Future research should validate these findings using additional datasets from different sources to confirm the generalizability of the results. Moreover, the practical implications of using SVM for claim frequency prediction, such as improving pricing accuracy and risk management, could be explored more thoroughly in applied insurance settings.
